# Fecundities of Hawaiian *Caenorhabditis briggsae* wild strains are not correlated with natural niche temperatures

**DOI:** 10.17912/micropub.biology.001356

**Published:** 2025-01-15

**Authors:** Nikita S. Jhaveri, Erik C. Andersen

**Affiliations:** 1 Biology, Johns Hopkins University, Baltimore, Maryland, United States

## Abstract

Adaptation to the local environment is crucial for organisms to survive and reproduce in the natural niche. The nematode *Caenorhabditis briggsae* has been studied extensively and hypothesized to be adapted to temperatures at the geographic sites where nematodes were isolated from nature. We empirically tested if this correlation is biologically meaningful by measuring the fitness of *C. briggsae* wild strains isolated from different temperatures across the Hawaiian islands. We did not observe a correlation between lifetime fecundity and the temperature of the natural niche from where the strains were isolated, indicating that this fitness trait might not be adapted to local environmental temperatures.

**Figure 1. Geographical location, ambient and substrate temperatures, and lifetime fecundities at four temperatures for the ten Hawaiian C. briggsae wild strains:**
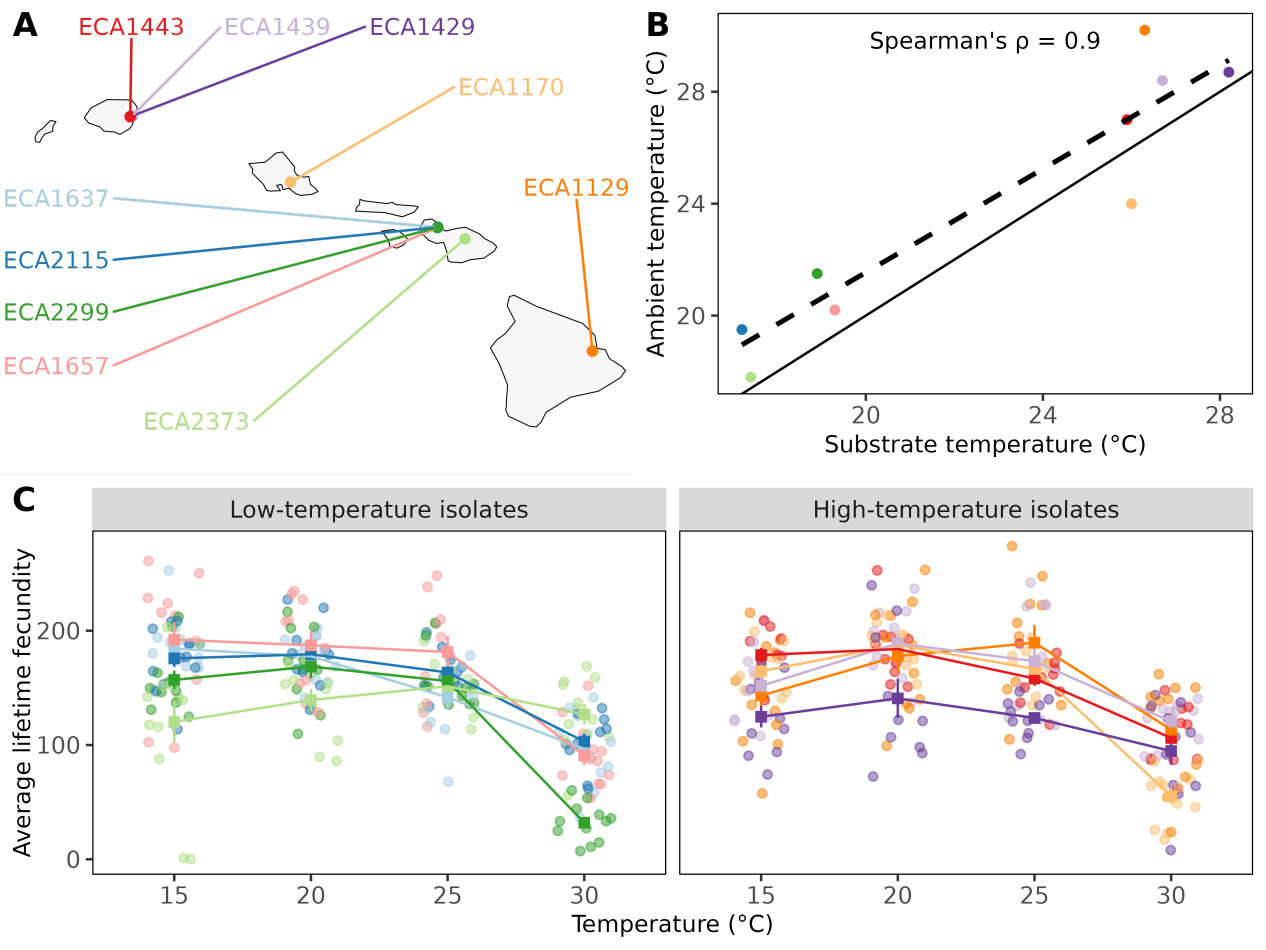
**A** . Geographical locations of the ten Hawaiian *C. briggsae* strains. **B.** Scatter plot showing a relationship between the ambient and substrate temperatures for each of the ten strains. Statistical analysis was conducted using a Spearman's rank correlation test (ρ = 0.902, *p* = 3×10 ^4^ ). The solid line indicates the reference line. The dashed line indicates the best-fit line. **C.** Lifetime fecundities of the ten strains at four different temperatures from 10 - 13 replicates were tested. No significant differences were observed between the low- and high-temperature isolates at 15°C, 20ºC, 25°C, or 30°C ( *p* = 0.067, 0.316, 0.872, and 0.305 respectively, Wilcoxon test).

## Description

Climate change is intensifying environmental pressures, compelling organisms to adapt rapidly to rising temperatures. Typically, species are finely tuned to specific niches where they thrive, but as global temperatures increase, these niches are shrinking, particularly in cooler regions. This reduction forces species to either adapt to warmer conditions or face extinction as cooler niches disappear. The loss of cooler habitats also leads to increased competition for the remaining suitable areas, further straining the survival of many species. Thus, climate change leads to not only alterations in the temperature but also causes restructuring of the ecological niches in which species survive.

Organisms adapt to particular niches using genetic mechanisms that allow them to thrive in specific environmental conditions. Instances of local adaptations are found in humans, plants, and animals. For example, human populations living near the equator have darker skin pigmentation compared to the ones from higher latitudes, protecting their skin from UV radiation (Jablonski and Chaplin 2010) . Plants from the desert have adapted to dry temperatures and have evolved mechanisms that help them minimize water loss (Mohanta et al. 2024) .

The nematode *Caenorhabditis briggsae* has been collected from various locations worldwide and has been clustered into distinct phylogeographic groups (temperate and tropical) based on the latitudes of isolation locations (Cutter et al. 2006; Félix et al. 2013; Crombie et al. 2019) . Previous studies suggested that strains are adapted to local climates using mortality or lifetime fecundity measures (Prasad et al. 2011; Wang et al. 2021) . Specifically, strains from temperate latitudes have higher lifetime fecundities than strains from tropical latitudes at cooler temperatures. By contrast, strains from tropical latitudes show higher lifetime fecundities than strains from temperate latitudes when raised at higher temperatures. Similarly, strains from the temperate regions are more resistant to cold than strains from tropical locations (Wang et al. 2021) . The classification of tropical and temperate clades is based on the latitudes from where *C. briggsae* strains were collected from nature and is not a measurement of the actual local temperature. In our study, we measured the temperature of the substrates at the time of isolation and measured the lifetime fecundities of *C. briggsae* strains at different temperatures.

To study local adaptation, we selected ten *C. briggsae* isotype reference strains isolated from the Hawaiian Islands ( Figure 1A ). Of the 75 strains that were collected from the Hawaiian islands, 46 strains had measurements of the substrate temperature at the time of isolation. We selected five strains that had been isolated from substrates with the lowest recorded temperatures and five strains that had been isolated from substrates with the highest temperatures ( Figure 1B ). Strains isolated from substrates with temperatures below 20ºC were designated "low-temperature isolates," and strains isolated from substrates with temperatures above 20°C were designated "high-temperature isolates". We hypothesized that, if the strains were adapted to their local environment, they would exhibit greater lifetime fecundities at temperatures closer to their substrate temperature, which could indicate the niche temperature. We tested this hypothesis at four temperatures: 15°C, 20°C, 25°C, and 30°C ( Figure 1C ). We found no correlation for lifetime fecundity between low- vs high-temperature isolates for all four tested temperatures (15°C, 20°C, 25°C, and 30°C; *p* = 0.067, 0.316, 0.872, and 0.305 respectively, Wilcoxon test).

Although we did not detect a correlation between lifetime fecundities and niche temperatures for the *C. briggsae* Hawaiian strains, several important caveats could explain this result. The most important caveat of our study is that we tested a small subset of the Hawaiian wild strains (ten isotype reference strains), and this sample might not be a true representation of the entire *C. briggsae* species. Second, the substrate temperature is not a true representative of the entire temperature range to which an individual *C. briggsae* strain might be adapted. The substrate temperature was recorded just at the time of substrate removal from nature. It is possible that *C. briggsae* strains are more labile and able to cope with a range of temperatures. Third, the conditions in the laboratory might not be able to recapitulate the wild niche, so lifetime fecundity measurements in the laboratory might not be reflective of differences found in nature. We also highlight that assigning clades based on latitude might not be reflective of genetic or environmental aspects of adaptation. Studies in plants have shown that different types of flavonoids are present in *Betula nana* leaves from northern Sweden and Alaska, both of which are located in similar latitudes (Jaakola and Hohtola 2010) . Another study showed that the soil temperature varies with altitudes in the same latitudinal location (Rodríguez et al. 2010) . These studies emphasize that latitude is a crude estimate of temperature preference, and local factors, such as elevation and temperature should instead be taken into consideration when studying local adaptation.

## Methods

Nematode culture maintenance

All strains used in this study are from the *Caenorhabditis* Natural Diversity Resource (CaeNDR) (Crombie et al. 2024) . Information about the samples is mentioned on CaeNDR. The animals were maintained at 20°C for three generations before starting the lifetime fecundity assay. Modified nematode growth medium (NGMA), which consists of 1% agar and 0.7% agarose was used to grow the strains on 6 cm plates (Andersen et al. 2014) . *Escherichia coli* strain OP50 was used as the food source.

Strain selection

All the isotype reference strains used in this study were isolated from the Hawaiian islands (Crombie et al. 2019; Crombie et al. 2022) . The ten strains chosen were based on temperatures of the substrates from which they were isolated. Of the ten strains, we selected five strains that had been isolated from substrates with the lowest recorded temperatures (low-temperature isolates), and five strains that had been isolated from substrates with the highest recorded temperatures (high-temperature isolates). The substrate temperatures mentioned are of the isotype reference strain.

Strains with their substrate temperatures and classification type (low- or high-temperature isolates) are:

ECA1637 (17.2°C, low-temperature isolate), ECA2115 (17.2°C, low-temperature isolate), ECA2373 (17.4°C, low-temperature isolate), ECA2299 (18.9°C, low-temperature isolate), ECA1657 (19.3°C, low-temperature isolate), ECA1443 (25.9°C, high-temperature isolate), ECA1170 (high-temperature isolate), ECA1129 (26.3°C, high-temperature isolate), ECA1439 (26.7°C, high-temperature isolate), ECA1429 (28.2°C, high-temperature isolate).

Lifetime fecundity assay

For each strain, 10 - 13 individual L4 larval stage hermaphrodites were picked on separate 6 cm OP50-seeded plates and incubated at 15°C, 20°C, 25°C, or 30°C. Each animal was transferred to a fresh plate every 24 hours until they stopped laying eggs. For the plates incubated at 15°C, animals were transferred up until day eight. For the plates incubated at 20°C, animals were transferred up until day five. For the plates incubated at 25°C and 30°C, animals were transferred up until day three. The plates with embryos were incubated at the same temperature as the hermaphrodite and allowed to grow until they were day-one adults before scoring to obtain daily fecundity (six days for 15°C, three days for 20°C, and two days for 25°C and 30°C). Lifetime fecundity for each replicate was calculated by summing the offspring from all the plates of the same replicate at the same temperature. If the hermaphrodite escaped, died, or could not be found on the plate after 24 hours, the replicate was discarded.

Statistical analysis

Statistical significance for lifetime fecundity at each temperature between low- and high-temperature isolates was determined using a Wilcoxon test. RStudio (v 4.2.1) was used to plot the data.

## Data Availability

Description: Raw data of lifetime fecundity . Resource Type: Dataset. DOI: https://doi.org/10.22002/ka9y4-z6948
